# Taking Stock: Assessing Animal Welfare in South African Captive Lion Tourism Venues

**DOI:** 10.3390/ani16172683

**Published:** 2026-08-27

**Authors:** Louise de Waal, Stephanie-Emmy Klarmann, Angie Elwin, Alexandra K. Schnell, Tom Moorhouse, Neil D’Cruze

**Affiliations:** 1 Blood Lions NPC, Gansbaai 7220, Western Cape, South Africa; management@bloodlions.org (L.d.W.); ncdcruze@gmail.com (N.D.); 2 Department of Psychology, University of Johannesburg, Johannesburg 2006, Gauteng, South Africa; 3 World Animal Protection, London EC2A 4NE, UK; angie.elwin@mmu.ac.uk (A.E.); alexschnell@worldanimalprotection.org (A.K.S.); 4 Department of Natural Sciences, Manchester Metropolitan University, Manchester M15 6BX, UK; 5 Department of Psychology, University of Cambridge, Cambridge CB2 3EB, UK; 6 Oxford Wildlife Research, Oxford OX4 3AS, UK; tompmoorhouse@protonmail.com

**Keywords:** African lion, animal–visitor interactions, captive felids, captive wildlife tourism, commercial captive breeding, *Panthera leo*

## Abstract

South Africa maintains thousands of captive lions and other felids in commercial captive wildlife facilities. Despite ongoing debate regarding the welfare implications of this industry, empirical assessments of welfare conditions remain limited. This study conducted a baseline assessment of welfare and husbandry conditions in public-facing captive lion venues in South Africa. A welfare assessment framework was applied across 34 tourism venues in Gauteng, Free State, Limpopo and North West provinces. Ten welfare indices were evaluated: mobility, environmental noise quality, shelter, hygiene, naturalness, social interaction, enrichment, diet quality, entertainment intensity, and animal management. Assessments were conducted using covert participant observation. Relationships between welfare scores, entry price and the provision of animal–visitor interactions (AVIs) were subsequently examined. A total of 627 lions were recorded across the 34 venues. No venue achieved high welfare scores across all indices, and welfare conditions were predominantly intermediate. Hygiene and environmental noise quality received the highest welfare scores, whereas animal management, enrichment, entertainment intensity and naturalness received the lowest. Recurring welfare concerns included restricted enclosure sizes, limited environmental complexity, inadequate shelter and retreat opportunities, inconsistent feeding practices, poor water quality, premature cub separation and intensive AVIs. Welfare scores were significantly higher at venues that prohibit AVIs than at venues offering such experiences. This study provides a standardised assessment of welfare conditions in South Africa’s captive lion tourism sector and contributes empirical evidence to the ongoing discussions regarding the regulation and future management of the industry.

## Simple Summary

South Africa has thousands of captive lions and other big cats kept in commercial wildlife facilities for tourists. While there has been considerable debate about the welfare of these animals, there is limited scientific evidence describing the conditions in which they are kept. This study provides a baseline assessment of welfare and husbandry standards at 34 public-facing captive lion tourism venues across four South African provinces. Using a standardised welfare assessment framework, we evaluated ten aspects of welfare, including enclosure quality, hygiene, shelter, environmental enrichment, diet, social opportunities, management practices and the intensity of tourist interactions. A total of 627 lions were assessed through covert observations. No venue achieved high welfare standards across all categories, and overall welfare was generally rated as intermediate. Hygiene and environmental noise were the strongest-performing areas, while animal management, environmental enrichment, naturalness and interactive activities received the lowest scores. Common welfare concerns included small enclosure sizes, limited environmental complexity, inadequate shelter, inconsistent feeding practices, poor water quality, early separation of cubs from their mothers and intensive animal–visitor interactions. Facilities that did not allow direct contact or interactions between visitors and lions consistently achieved higher welfare scores than those that offered these activities. These findings provide important evidence to inform policy and regulation for South Africa’s captive lion tourism industry.

## 1. Introduction

Big cats (*Panthera* spp.) are iconic, charismatic species and represent some of the most threatened taxa globally [1]. The African lion (*Panthera leo*) has disappeared from approximately 92% of its historic range, and the population declined by approximately 43% between 1993 and 2014, leaving an estimated 23,000 individuals remaining in the wild [2]. Consequently, the species is currently listed as “Vulnerable” on the International Union for Conservation of Nature (IUCN) Red List [3,4]; however, wild lion populations in South Africa are currently considered stable [5].

Concurrently, substantial numbers of captive-bred lions are kept in private commercial facilities in South Africa [6]. In 2023, a national audit identified 342 facilities across eight of South Africa’s nine provinces holding Threatened or Protected Species (TOPS) permits, collectively housing at least 7838 captive lions and more than 2315 other carnivores [7]. Other felids maintained in captivity for commercial purposes include leopard (*Panthera pardus*), cheetah (*Acinonyx jubatus*), caracal (*Caracal caracal*), serval (*Leptailurus serval*), and non-indigenous species like tiger (*Panthera tigris*), jaguar (*Panthera onca*) and puma (*Puma concolor*) [7].

In South Africa, lions have been bred for the captive hunting industry since the 1990s [8], with trophy exports peaking in 2016, shortly after which the United States Fish and Wildlife Service imposed a ban on imports of trophies from captive-bred lions [9]. Despite this restriction, trophy hunting of captive-bred lions has persisted in subsequent years, with South Africa exporting on average just over 400 captive-bred lion trophies per year [10,11].

From 2008, increasingly large volumes of lion skeletons were also exported from South Africa, either as a by-product from captive hunting or as a primary product, with lions bred specifically for their bones [12]. Demand was driven predominantly by traditional medicinal and cultural practices in Southeast Asia, particularly the production of medicinal and tonic wines that traditionally utilise tiger bones [13,14]. Between 2008 and 2016, more than 6000 lion skeletons, mostly from the captive-bred population, were exported from South Africa [15] to supplement and, in some cases, substitute for tiger bones used as ingredients in traditional medicine [14,16]. In 2017 and 2018, annual CITES export quotas of 800 lion skeletons were introduced; however, these quotas were rescinded in 2019 by a High Court judgement that deemed them “unlawful and constitutionally invalid” for failing to consider animal welfare [17]. Since then, the Department of Forestry, Fisheries and the Environment (DFFE) has deferred setting further CITES export quotas [10].

Indigenous and non-indigenous felids are also widely used within South Africa’s captive wildlife tourism sector. An estimated 74 tourism venues hold lions, cheetahs and/or tigers [18], many offering animal–visitor interactions (AVIs). These facilities use a variety of titles, including “reserves”, “predator parks”, “sanctuaries” and “farms”, and their stated purposes vary considerably. Claimed objectives range from providing welfare benefits (e.g., housing “rescued” or “orphaned” animals) and educational value, to conservation initiatives, alongside the explicit breeding of lions for captive hunting [19].

In summary, three recognised revenue streams have arisen from the breeding and keeping of captive lions: (1) the tourism and voluntourism sector (in which travellers engage in a working holiday, volunteering their time for charitable causes [20]; (2) the captive trophy hunting industry; and (3) the trade in live lions, their bones, parts and derivatives [12,21]. Individual lions may remain within a single sector throughout their lives, but they can also be moved between sectors at different stages of their development [10]. For example, lion cubs that become too old for safe use in AVI are often moved or sold into lifelong captivity for breeding or exhibition, traded live, or killed for captive trophy hunts or the trade in their bones, parts, and derivatives [22,23]. Furthermore, commercial facilities frequently operate with parallel legal entities with public-facing and fully off-grid business components, the latter generally undisclosed and inaccessible to the paying public [24]. They showed that some facilities may use legal operations to practise illegal activities, including illegal international trade in lion bones [24].

The captive lion industry is contentious for several reasons. Although many stakeholders claim that lion breeding provides conservation benefits [19], there is little to no evidence supporting any meaningful conservation value [19,25,26]. Furthermore, in South Africa the lack of a safe and suitable habitat for rewilding and restoration purposes, and the relatively high availability of wild lions from the metapopulation, makes the use of captive-bred lions for conservation effectively redundant [10,27]. Some studies have also suggested that the industry may pose threats to wild lion and tiger populations by sustaining or stimulating demand for big cat products, creating opportunities for the laundering of wild-sourced specimens into legal markets, and potentially incentivising the targeted poaching of wild individuals [2,3,4,14,26,28,29]. Furthermore, the industry remains largely unregulated [30,31] and has been linked to illegal international wildlife trade [6,14,24]. Potential risks relating to zoonotic disease transmission, to tourists and staff, for example, have also been identified [32]. In addition, several publications and policy reports have suggested that the industry may negatively affect South Africa’s tourism and conservation reputation [27,33,34]. Finally, numerous studies have raised animal welfare concerns associated with the breeding, keeping and trading of lions and other felids in captive environments [22,35].

Animal welfare concerns associated with captive lion facilities have been highlighted in the National Council of Societies for the Prevention of Cruelty to Animals (NSPCA) inspection reports and documented case studies from South Africa. For example, in 2022 the NSPCA, the statutory body responsible for enforcing the Animals Protection Act (APA), 71 of 1962, inspected 127 captive lion facilities. These inspections resulted in 21 warrants, 49 warnings, six notices, eight dockets, 17 people accused of animal welfare violations, and two court cases [36]. The NSPCA reported that basic welfare standards were often lacking, including inadequate shelter and nutrition, lack of clean water, poor hygiene, overcrowding, absence of environmental enrichment and insufficient veterinary care [36]. Concerns about poor conditions in captive facilities have also been highlighted in the media, providing illustrative examples of persistent welfare issues. These include reports of emaciated lions in Limpopo [37]; overcrowding and disease (mange and meningoencephalitis) in the North West [38]; and two separate incidents in the Free State: 30 lions with severe burns [39] and a leopard confined in a trailer for five months [40].

With respect specifically to lion AVI, studies have documented a range of welfare concerns, including lion cubs subjected to inappropriate social grouping, the inability to retreat from forced interactions, insufficiently trained and dedicated caretakers and poor breeding practices [22]. An assessment of 49 YouTube videos depicting lion cubs used for AVI experiences across 11 South African captive wildlife venues identified poor husbandry practices, with an average of four issues per video [23]. These included the use of cubs under three months without their mother present (61% of videos) and daytime AVI activities occurring during periods when cubs would typically be inactive. Stress-related behaviours, most commonly avoidance or aggression, were observed in 77% of the videos [23]. Furthermore, public-facing captive wildlife venues are often considered “sanitised” compared to commercial off-grid lion facilities [24,41]. As such, welfare conditions in off-grid commercial predator facilities may be comparable or potentially poorer.

The welfare of captive wild animals falls within the scope of South Africa’s APA, which aims to prevent cruelty and neglect, or “unnecessary suffering”. Since the 2019 High Court ruling, the National Environmental Management: Biodiversity Act (NEMBA), 10 of 2004, has seen the inclusion of an animal well-being clause in 2023. Under this provision, animal well-being means “the holistic conditions conducive to an animal’s physical, physiological, and mental health and quality of life, including its ability to cope with its environment”. This marks an important shift in wildlife management and governance, and applies to both free-roaming and captive wild animals.

While these legislative developments represent an important step towards recognising the well-being of captive wild animals, their effective implementation remains challenging. Systematic monitoring and enforcement of welfare standards within South Africa’s commercial captive lion industry continue to face severe limitations. Provincial legislation governing captive lions is inconsistent across South Africa’s nine provinces [10], creating legal loopholes [30]. Enforcement is undertaken by provincial conservation authorities that are widely reported to lack sufficient staffing and resources [27] and welfare expertise [10]. The NSPCA operates independently of state funding and repeatedly encounters accessibility challenges in carrying out its welfare mandate [10,30]. While several stakeholder organisations have developed voluntary standards and guidelines for captive lions [42,43,44], these are non-binding and lack enforcement mechanisms. Tourists may also have limited capacity to evaluate husbandry conditions and the welfare implications of captive wildlife use [19], making it unlikely that consumer behaviour alone can exert meaningful regulatory pressure through market feedback mechanisms [19,45,46].

Consequently, despite growing legal recognition of animal well-being, the welfare state of captive lions and other felids in South Africa’s commercial wildlife industry remains largely unknown [47]. In the absence of national norms and standards, there is a need for standardised and objective welfare assessment approaches. Building on this need, this study conducted a baseline assessment of welfare and husbandry conditions for captive lions in South African tourism venues using a practical scoring framework adapted from Schmidt-Burbach et al. [48]. By applying a consistent assessment methodology across venues, this study aimed to obtain empirical evidence relevant to understanding conditions within the wider commercial captive lion industry and informing ongoing policy discussions, including the South African government’s 2021 commitment to phase out the commercial captive lion industry [49].

## 2. Methodology

### 2.1. Sample and Study Area

A total of 39 tourism venues were identified across Gauteng, Free State, Limpopo and North West provinces, South Africa (Table 1). These provinces were selected to capture variation in regulatory contexts and because Free State, Limpopo and North West are the primary regions associated with the commercial captive lion industry [7]. Venues were selected using purposive criteria including: (i) accessibility to the public during regular visiting hours; (ii) the presence of captive lions used for entertainment purposes, including viewing and/or AVI experiences; and (iii) a location within an hour’s drive of major tourism routes originating from OR Tambo International Airport, Johannesburg.

Five venues were excluded as they were closed at the time of the research visits. The final sample therefore comprised 34 venues, including nine surveyed during a pilot phase and 25 in the main assessment.

The pilot study was conducted in May 2023 by a team of four researchers affiliated with two animal welfare organisations. During this phase, nine tourism venues located in Gauteng, Limpopo, and North West provinces were assessed. The pilot aimed to test the feasibility and clarity of the welfare assessment framework. Following this phase, minor revisions were made to improve the clarity and consistency of wording in the assessment forms prior to deployment in the main assessment.

The main assessment was conducted over a two-week period in March 2024 during South Africa’s off-peak tourist season. Independent fieldworkers with experience in environmental and animal welfare investigations conducted the assessments. Two teams, each comprising two to three researchers, were deployed to assess the remaining 25 tourism venues across Gauteng, Limpopo, Free State and North West provinces.

Visits were conducted across different days of the week during the off-peak tourist season and may not fully represent operational conditions during peak visitation periods. For example, visitor numbers, staffing patterns, and the frequency of AVI may differ seasonally.

### 2.2. Data Collection

Data were obtained through: (1) structured observations guided by predefined criteria using a welfare assessment framework, (2) the collection of photographic and video evidence, and (3) informal discussions with venue staff.

Data collection was limited to publicly accessible areas and information available to visitors through observations, staff interactions and on-site signage. Consequently, the dataset reflects only conditions observable to the public and did not capture animals housed in non-public areas or practices not disclosed by staff.

Additional information regarding venue details, biosecurity and safety provisions, and informational resources was collected using the Venue Assessment Form (Supplementary File S1, page 1). This included basic data pertaining to the availability and quality of informational resources for visitors on species, conservation and animal welfare, as well as the provision of hand sanitizer, personal protective equipment, safety barriers for predator viewing and safety measures for AVI.

Venues were coded in a database and names and locations were excluded to further protect study participants from harm or discrimination according to ethical guidelines [50].

#### 2.2.1. Welfare Assessment Framework

The welfare scoring assessment evaluated the welfare and husbandry conditions of captive lions in tourism venues open to the public. Data were collected using a covert participant observation approach, whereby researchers accessed facilities anonymously as paying visitors. This approach has previously been used to document welfare conditions of captive animals in tourism settings [26,51].

The assessment framework incorporated criteria adapted from Schmidt-Burbach et al. [48], modified to reflect lion biology and relevant South African regulatory contexts (Supplementary File S2). The framework included ten lion welfare indices scored on a five-point scale (0–4): mobility, environmental noise quality, shelter, hygiene, naturalness, social interaction, enrichment, diet quality, entertainment intensity, and animal management (Table 2).

Although the primary focus was on lion, the framework also recorded data on other indigenous and non-indigenous felids present at each venue (Supplementary File S3). Observations were recorded on standardised welfare assessment forms during each visit (Supplementary Files S2 and S3).

#### 2.2.2. Collection of Photographic and Video Evidence

Photographic and video evidence was collected during visits to document enclosure conditions, animal behaviour, environmental features, husbandry practices and AVI observable to the public. These materials were collected to supplement structured field observations, enable retrospective review of welfare conditions and support consistency in scoring between researchers.

Supplementary trip reports were also compiled to capture contextual observations beyond the structured assessment framework.

#### 2.2.3. Informal Staff Interactions

Additional contextual information was obtained through informal and unstructured interactions with venue staff during the visits. These discussions provided information and insights into husbandry practices, feeding regimes, animal management and operational procedures. No formal interviews were conducted.

### 2.3. Data Analysis

#### 2.3.1. Quantitative Welfare Assessment

Field researchers compiled observational data, as well as photographic and video evidence collected during venue visits into structured spreadsheets organised according to venue characteristics, lion welfare, enclosure conditions, other species, other felids and field notes. All data were anonymised.

The ten lion welfare indices were scored on a 0–4 scale for each venue (Table 1). Scoring was conducted independently by two researchers with expertise in captive predator welfare using triangulation across field notes, photographs, video recordings and assessment forms to corroborate findings and support interpretive consistency.

Following independent scoring, discrepancies between researchers were reviewed collaboratively and adjusted where necessary to ensure that the final scores accurately reflected observed welfare conditions. Where welfare conditions varied substantially within facilities (e.g., between cub and adult enclosures), scores were weighted toward the poorest conditions observed.

#### 2.3.2. Qualitative Analysis

Qualitative welfare data were synthesised from two primary sources: (1) structured assessment forms completed during field visits, and (2) systematic reviews of photographic and video material collected at each venue. Data were coded deductively within predefined welfare categories derived from the assessment framework.

Photographic and video materials were reviewed using a structured annotation process, with observations coded according to the type of welfare concern, strength of evidence and relevant welfare category. Both behavioural and environmental indicators were documented alongside staff-reported management practices where these were directly observed or explicitly stated during visits.

#### 2.3.3. Statistical Analysis

Variability in the ten lion welfare indices was examined across the 34 venues and assessed in relation to venue characteristics observable to tourists, including entry price and the provision of AVI activities. Summary statistics were calculated for all welfare index scores.

To assess the potential relationships between welfare conditions and venue characteristics, the ten welfare indices were combined into a single composite measure of lion welfare using exploratory factor analysis (EFA), a statistical method used to reduce correlated variables into a latent construct representing the overall welfare condition [52,53].

The EFA was conducted in R (version 4.5.2) using the Factanal package with varimax rotation. Factor scores were extracted and used as the dependent variable in subsequent linear models. Entry price was included as a continuous predictor.

To examine whether lion welfare differed according to the provision of AVI, the same analytical approach was repeated using a modified index set excluding “entertainment intensity,” as this variable directly captured the presence of AVI and would otherwise introduce circularity into the model. Model assumptions, including normality of residuals, homoscedasticity and linearity, were assessed and met.

### 2.4. Ethical Considerations

Covert participant observation was employed to assess routine operational conditions while minimising observer effects that could alter husbandry practices or the presentation of animal welfare conditions. All data were collected by researchers accessing venues lawfully as paying members of the public and were limited to information ordinarily available to visitors. Venue identities and locations were anonymised throughout the study, while employee details were not gathered during data collection to ensure their privacy. Information obtained through informal discussions with venue staff is reported only in anonymised form where it provides contextual information relevant to the interpretation of the observational findings. Photographs included in this manuscript were reviewed prior to publication to avoid identifiable features. This maintained venue anonymity while illustrating representative welfare conditions to minimise potential harm to participating venues and individuals.

## 3. Results

### 3.1. Captive Lions

#### 3.1.1. Overview of Venues and Numbers of Lions

**Figure 1:**
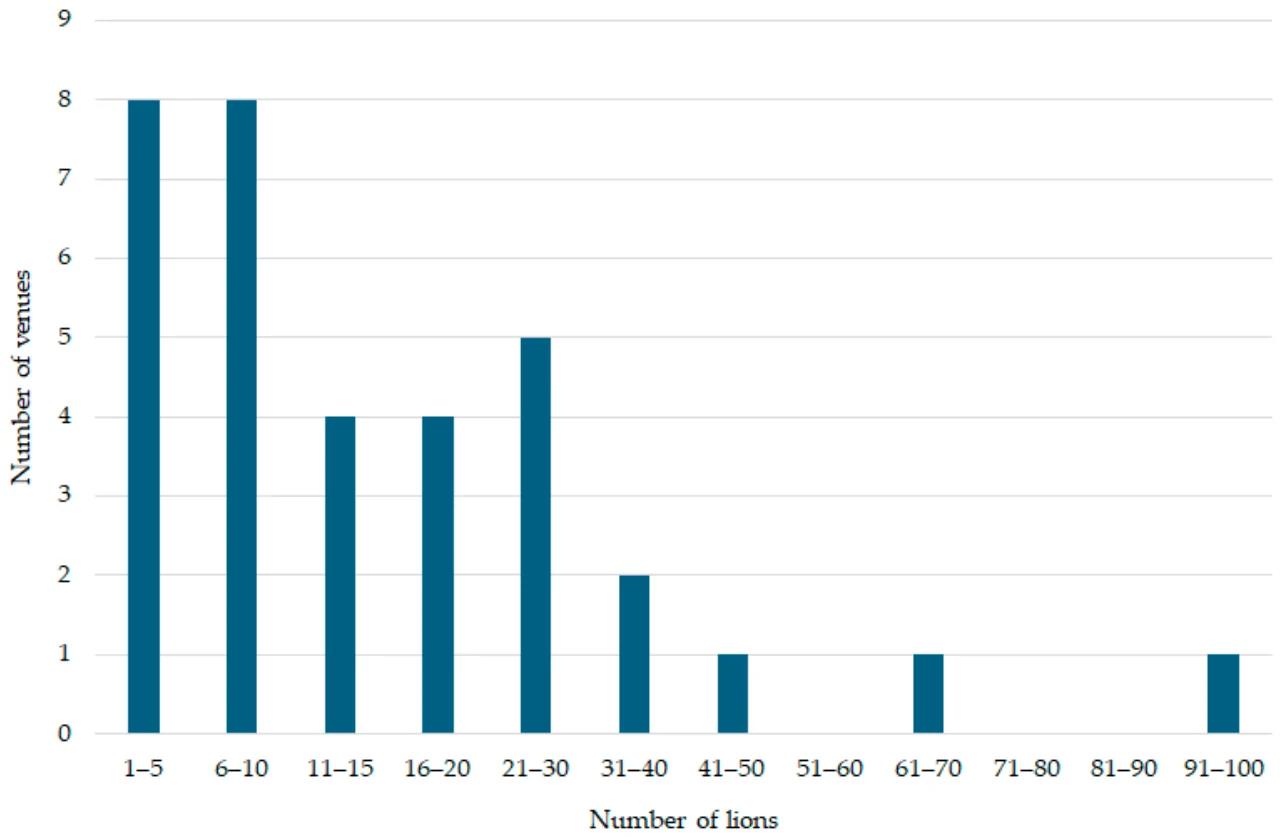
The distribution of captive lions held across the 34 venues assessed in South Africa.

Across the 34 venues assessed, a total of 627 lions (including both adults and cubs) were recorded. The distribution of lions across venues was skewed, with a small number of facilities housing disproportionately large populations. Three venues housed 50 or more lions each, including one with approximately 100 individuals (Figure 1). More than half of the venues (58.8%, *n* = 20) housed 15 or fewer lions, accounting for 22.0% (*n* = 138) of the recorded number of captive lions, while the remaining 41.2% of venues (*n* = 14) housed 78.0% (*n* = 489) of the captive lions in the study (Figure 1). The sex ratio of lions was skewed toward females (60.7%). Of the 627 lions recorded, 16.8% were white lions (*n* = 105 across 22 venues), 1.9% were hybrids (*n* = 12 across six venues) and 8.3% were cubs at the time of the assessment (*n* = 52 across 12 venues).

Based on information provided by venue staff, 20.6% (*n* = 7) of the venues (V5, V9, V12, V26, P7, P8 and P9) operated parallel entities with a public-facing side and an off-grid business component that is not accessible to paying visitors. According to the information disclosed by venue staff, off-grid business components included breeding for live domestic and international trade; however, captive hunting and trade in parts and derivatives were not freely disclosed.

#### 3.1.2. Entry Price, Types of Tourist Experience Offered, and Interaction Intensity

Entry costs were obtained for 27 venues, with the most common fee range of ZAR100–250 per adult (66.7%, *n* = 18). The remaining venues charged under ZAR100 (11.1%, *n* = 3), ZAR250–500 (11.1%, *n* = 3), and more than ZAR500 per adult (11.1%, *n* = 3).

Tourism models varied, with 20 venues offering on-demand access and 14 operating based on scheduled tours. Visitor experiences included guided tours (*n* = 22); self-guided tours (*n* = 5); walk through experiences (*n* = 13); and game drives (*n* = 4), with some venues offering more than one type of experience.

Venues differed markedly in terms of AVI offerings with lions. Twelve venues (35.3%) did not offer any AVI with lions at the time of the study, with two explicitly stating a policy against AVI. Cub entertainment was offered most frequently with 32.3% (*n* = 11) offering cub petting, 26.5% (*n* = 9) offering selfies with cubs, and 5.9% (*n* = 2) offering cub feeding (Table 3a). Animal–visitor interactions with adults included walking with lions (8.8%, *n* = 3), selfies with lions (14.7%, *n* = 5), and guides provoking lions for entertainment (23.5%, *n* = 8) or encouraging lions to perform tricks (2.9%, *n* = 1) (Table 3a).

#### 3.1.3. Information Provided for Tourists

Among the 34 venues, 52.9% (*n* = 18) provided varying levels of lion species information, 32.4% (*n* = 11) provided information on lion conservation, and 29.4% (*n* = 10) provided information on animal welfare (Table 3b). Conversely, 47.1% (*n* = 16), 67.5% (*n* = 23) and 70.6% (*n* = 24) of venues, respectively, provided no information on these topics (Table 3b). Where information was provided, it was typically basic, e.g., posters or signage, rather than comprehensive, e.g., compulsory video, dedicated staff, or presentations (Supplementary File S1). Comprehensive information was only provided by 20.6% (*n* = 7) venues on lion species, 17.6% (*n* = 6) on conservation, and 8.8% (*n* = 3) on animal welfare (Table 3b).

#### 3.1.4. Biosecurity and Safety Standards

Biosecurity measures to mitigate against any potential transmission of zoonotic diseases were generally limited. Hand sanitisers were observed at four venues (11.8%) and absent at 24 (70.6%), with the situation being unknown at the remaining six venues (17.6%) (Table 3c, Supplementary File S2). Personal protective equipment (PPE) and dedicated footwear were not observed at any of the venues and were recorded specifically as absent at 61.8% of the venues (*n* = 21). Disinfection procedures on entry to or exit from the venue and/or enclosures were observed at 8.8% of venues (*n* = 3) and were optional at 2.9% (*n* = 1), while 88.2% of venues did not provide such measures (*n* = 17) or it was unknown (*n* = 13) (Table 3c).

The provision of stand-off barriers in front of enclosure fencing preventing contact with the lions varied considerably. Five venues (14.7%) had such barriers for both staff and visitors and 35.3% of venues (*n* = 12) had stand-off barriers for visitors only—this included those venues that offered vehicle-based tours or self-drives, where safety guidelines instructed visitors to remain in vehicles. The remaining 50% of venues (*n* = 17) had no barriers for either staff or visitors.

### 3.2. Lion Welfare Indices

#### 3.2.1. Overall Welfare Scores

**Figure 2:**
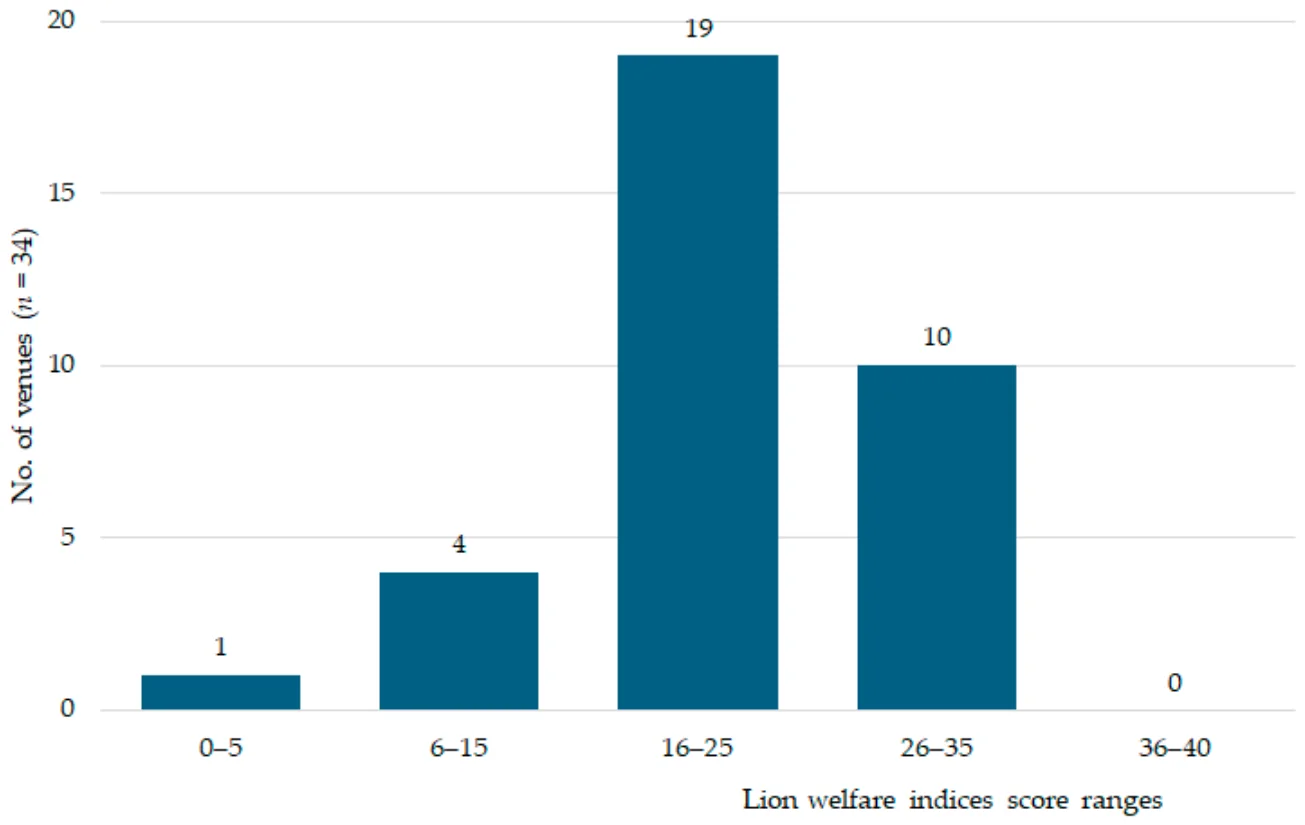
Overall score ranges of ten lion welfare indices across 34 venues assessed in South Africa.

The maximum welfare score achievable per venue was 40 points. Of the 34 venues assessed, the most notable results were that more than half (55.9%, *n* = 19) scored within the intermediate range of 16–25 points, only one venue (2.9%) scored fewer than 5 points, and no venues scored above 35 points (Table 2; Figure 2).

**Figure 3:**
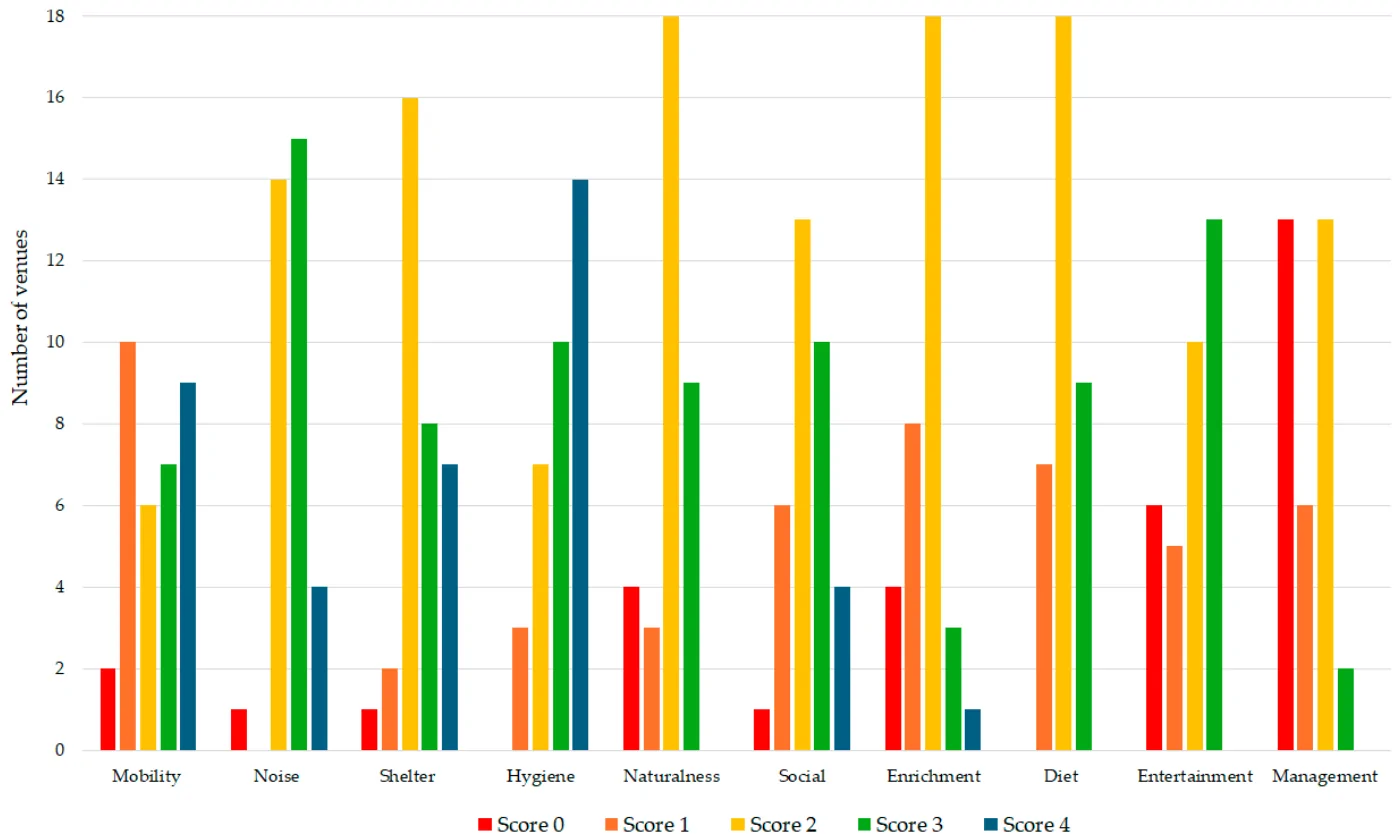
Scores across the ten lion welfare indices for the 34 tourist venues assessed in South Africa (scores for all welfare indices are described in Table 2).

Scores across the ten lion welfare indices for the 34 tourism venues assessed were predominantly within the intermediate range, with most indices averaging a score of 2.0 (Figure 3). Hygiene received the highest overall average score (3.0), followed by environmental noise quality (2.6). In contrast, the lowest average scores were recorded for animal management (1.1), enrichment (1.7), entertainment intensity (1.9) and naturalness (1.9). The remaining welfare indices (mobility, shelter, social interaction and diet quality) also fell within the intermediate range, with average scores of 2.3, 2.5, 2.3 and 2.1, respectively.

#### 3.2.2. Welfare Findings for Lions Across All Indices

In addition to the quantitative welfare index scores provided in Table 2 and Table A1 across the 34 venues, a qualitative analysis of observational, photographic and video evidence, and staff reported information, identified consistent patterns across all ten welfare indices, although the conditions varied between facilities.

##### Mobility

Mobility scores ranged widely between zero and four (Table 2) with 5.9% venues scoring zero (*n* = 2), 29.4% scoring 1.0 (*n* = 10), 17.6% scoring 2.0 (*n* = 6), 20.6% scoring 3.0 (*n* = 7), and 26.5% scoring 4.0 (*n* = 9) (Figure 3). Venues receiving the highest mobility score provided enclosures with a minimum of 5000 m^2^ per lion.

Qualitative observations further highlighted substantial variations in enclosure size and functional space relative to age class and group size. Cubs were frequently housed in small indoor or partially covered holding areas (e.g., V5, V6 and V10) and even in small crates (e.g., V17). In many enclosures, limited shade or microhabitat availability appeared to further reduce the amount of usable space available to lions, with individuals often clustering around a single shaded area rather than utilising the enclosure more broadly (e.g., V5 and V18). In contrast, some facilities provided larger, more naturalistic enclosures with greater spatial complexity and opportunities for dispersal (e.g., V12, V23 and V29). Although certain venues provided corridors or adjoining spaces intended to increase access to larger areas (e.g., V12), these did not consistently correspond with greater movement or use of space in the observed footage. Overall, smaller and structurally simple enclosures were frequently associated with reduced opportunities for lions across age classes to move freely, distance themselves from the public or conspecifics, and engage in behaviour that approximates species-typical ranging behaviours.

##### Environmental Noise Quality

Environmental noise quality scores were generally in the intermediate to high range, varying between two and four (Table 2 and Figure 3). One venue (2.9%) received a score of zero due to the presence of large crowds and electronic noise from a loudspeaker, while no venues received a score of one, 41.2% of venues (*n* = 14) scored two, 44.1% (*n* = 15) scored three, and 11.8% venues (*n* = 4) scored four. Overall, just over half of the venues (*n* = 19) scored three or four, indicating that lions were predominantly exposed to natural sounds in the animals’ environments.

Qualitative observations indicated that noise exposure differed across venues and was largely associated with visitor activity and operational practices. Recorded sources of noise pollution included tour groups, vehicles operating close to enclosures, and in some cases amplified music in visitor areas. Continuous generator noise operating near lion enclosures was also recorded, creating sustained background noise likely to limit opportunities for undisturbed rest (e.g., V18, V22 and V26). Some guides were observed using sharp auditory cues, such as shouting or striking enclosure structures, introducing intermittent sudden noise events and the use of amplifiers during guided tours (e.g., V8, V10 and V12).

##### Shelter

The provision of shelter varied considerably across the 34 venues, but scores were primarily within the intermediate range (47.1%, *n* = 16, score 2) (Table 2 and Figure 3). Only 2.9% of venues (*n* = 1) scored zero and 5.9% (*n* = 2) scored one due to a lack of external shelter and barren or concrete substrates. In contrast, 23.5% of venues (*n* = 8) scored three and 20.6% (*n* = 7) scored four, indicating the provision of reasonably natural ground substrate and adequate shelter.

Qualitative observations indicated that shelter provision was inconsistent across venues. In the lower scoring venues, shelter provision was inadequate with some venues offering basic platforms and roofing (e.g., V1, V6 and V19). With over half of the venues scoring 2 or below, many enclosures lacked dens, visual barriers or adequate shade, leaving animals exposed to the elements and with limited opportunities to withdraw from conspecifics or visitor presence. Several enclosures provided no natural shade at all, or only a single small tree beneath which lions clustered (e.g., V5 and V18). Across multiple venues, shelters appeared unstable, weathered or minimally protective, and at one venue lions relied on fallen logs rather than purpose-built shelters for respite (e.g., V22). Less than half of the venues offered adequate tree cover or simple but functional shelters (e.g., V2, P6 and V13). Overall, retreat options (areas where animals can withdraw from public view or avoid conspecifics) and weather protection were not reliably available across all venues.

##### Hygiene

Hygiene scores were primarily high across the venues, with 70.6% of the venues (*n* = 24) receiving a score of three or four, indicating frequent cleaning and the absence of old food remains and faeces (Table 2 and Figure 3). No venues received a zero score, 8.8% (*n* = 3) received a score of one, and 20.6% of the venues (*n* = 7) received an intermediate score of two due to the presence of recent food remains and faeces, which precluded higher scores.

Qualitative observations identified hygiene concerns at several venues. Video footage and photographs from these venues documented green or murky drinking water (e.g., V25), and accumulation of old bone and carcass remains (e.g., V13). At one venue (V5), a guide stated that some enclosures are only cleaned every few months due to safety concerns. Another venue (V22) showcased lion enclosures containing hundreds of defrosted chicken carcasses, with limited evidence of consumption by the lions. Although most venues (91.2%, *n* = 31) scored two or above, the lack of routine cleaning and water management at some venues were not just basic animal welfare concerns but had added implications for disease risk and environmental contamination. Higher scoring venues (e.g., P1. V11 and V28) demonstrated clean water sources and enclosures with an absence of old bones, leftover meat and accumulated faeces.

##### Naturalness

More than half of the naturalness scores were in the intermediate range with scores of two (52.9%, *n* = 18), indicating some natural surroundings but predominantly artificial structures in the lions’ immediate vicinity (Table 2 and Figure 3). One in five venues (20.6%, *n* = 7) scored zero or one, reflecting environments that were largely urban, barren or fully artificial. Although 26.5% (*n* = 9) of venues scored three, no venues scored four because none of the assessed environments resembled a functioning ecosystem.

Qualitative observations showed that enclosure environments ranged from barren camps to moderately vegetated areas with trees, shrubs, rocks and occasional pools, although structural complexity was generally limited (e.g., P8, V19 and V21). Even where more natural features were present, the ecological functionality of these spaces was limited, constraining opportunities for exploration, thermo-regulation and species-typical behaviours (e.g., V20).

The naturalness of cub enclosures was generally poorer than those of the adult lions. Some venues housed cubs in enclosures with virtually no natural features, consisting primarily of hard mud substrate with minimal vegetation or environment complexity. In two cases (V6 and V22), cubs were housed in the front yard of a dwelling without any natural elements.

##### Social Interaction

Social grouping was managed by human intervention and varied considerably across the 34 venues (Table 2 and Figure 3). Social interaction scores for 38.2% (*n* = 13) of venues were within the intermediate range (score two), reflecting dense group housing and limited opportunities for lions to retreat from the public or conspecifics. One in five venues (20.6%, *n* = 7) scored zero or one, indicating overcrowding, solitary lions, inappropriate sex ratios and/or no visual barriers between conspecifics. Less than half of the venues (41.2%, *n* = 14) scored three or four, indicating greater opportunities for lions to regulate social interactions.

Qualitative observations showed that lions in lower-scoring venues were sometimes housed solitarily (e.g., P2 and P8), while mixed-species housing, including lions and tigers across different age classes, was also recorded (e.g., V5 and P9). Venues with higher scores had less dense group housing and increased space to allow conspecifics to retreat from each other (e.g., P1. V2 and P6).

Cubs were frequently separated from their mothers at one to three weeks of age for bottle-rearing, eliminating opportunities for maternal care and natural pride interactions. Staff at some venues reported maternal distress following removals (e.g., V5 and V6). Cubs were routinely handled by staff or presented for AVI, including petting, bottle-feeding and photography (e.g., P9). Large numbers of cubs at some facilities suggest sustained breeding to maintain a continuous supply of young animals for AVI. A small number of venues stated they had reduced or even ceased breeding of lions and hand-raising of cubs (e.g., V21, V23 and V24).

##### Enrichment

More than half of the venues scored within the intermediate range for enrichment (52.9%, *n* = 18 score 2) (Table 2 and Figure 3). This reflected the presence of some enrichment, although it was generally limited in terms of variety or the frequency of updating enrichment. A score of zero or one was recorded for 35.3% (*n* = 12) of venues, indicating an overall lack of enrichment and even vertical structures. Only 11.8% of venues (*n* = 4) received a score of three or four.

Qualitative observations indicated that purposeful enrichment was rare. Most facilities offered only basic features such as logs, platforms, access to a water source or a ball, and these were often insufficient to provide sustained cognitive or behavioural stimulation (e.g., V1, P2, P8 and P9). One venue (V29) provided a more nature-based enclosure with trees, rocks and ponds, therefore enabling more enriching activities for adult lions. Cub areas occasionally included toys or small objects, yet the surrounding environments remained largely barren and lacked the complexity needed to support natural behavioural development (e.g., V5, V26 and V28). Overall, enrichment tended to be simple, inconsistently applied, and largely insufficient to provide lions with diverse, species-typical behavioural opportunities.

##### Diet Quality

Diet quality scores were primarily within the intermediate range, with no venues receiving either the lowest or highest scores (zero or four) (Table 2 and Figure 3). More than half of the venues (52.9%, *n* = 18) received a score of two, while the remaining venues scored either one (20.6%, *n* = 7) or three (26.5%, *n* = 9). This means that the majority of venues provide food in adequate amounts but limited in variety and inappropriate delivery.

Qualitative observations indicated substantial variation in feeding practices. Some venues provided cubs with milk formula and supplements (e.g., V22), while verbal reports of food shortages for cubs were also documented (e.g., V5). Diets for sub-adult and adult lions ranged from a red meat diet (e.g., P4) to relying heavily on chicken, with feeding times in some cases scheduled to coincide with tourist viewing times, or inconsistent feeding schedules (e.g., V12 and V21). Both under- and overweight lions were observed across the 34 venues (e.g., V1. V20. V21, V24 and V27). In some cases, large quantities of defrosted chickens were observed in the enclosures with minimal interest from the animals (e.g., V20 and V22). These findings highlight highly inconsistent nutritional practices that contributed to visible disparities in body condition and overall welfare. As noted under hygiene, there was evidence of green and murky water in some venues, indicating that clean water replenishment was infrequent.

##### Entertainment Intensity

Entertainment intensity scores varied across the 34 venues, although no venues scored four indicating that all offered some level of AVI (Table 2 and Figure 3). A zero score was recorded for 17.6% (*n* = 6) of venues, and 14.7% (*n* = 5) scored one, indicating that one-third offered regular and intense commercial entertainment. The remaining venues scored two (29.4%, *n* = 10) or three (38.2%, *n* = 13), indicating that although commercial entertainment was more limited, lions were generally still unable to withdraw voluntarily from AVI activities.

Qualitative observations documented AVI across most venues, including cub petting, cub feeding, selfies, walking with lions and guide-facilitated interactions. Visitor numbers during these activities were high at some venues, with approximately 6–20 tourists observed participating in cub handling or walking interactions during the low tourism season (e.g., V10, V26 and V30), while other venues stated that they can receive up to 400 visitors per day during the peak season (e.g., V24). For example, video footage showed tourists positioned around cubs with staff carrying and placing cubs for handling (e.g., V10 and V26). Close-contact walking with sub-adult and adult lions was observed, along with the use of sticks or verbal cues to keep animals moving or to stage particular poses for photographs (e.g., V10 and V30). At one venue, visitors were encouraged to walk behind adult males, pose in close proximity with their backs turned to the lions, and/or lions were prompted to climb or sit on command for photographs (e.g., V30). These AVI practices prioritise visitor experience and introduce clear risks of stress and compromised safety for both animals and people.

Venues P1, P2, and P4 demonstrated less intense entertainment experiences; however, it should be noted that the visits were conducted on weekdays and may not reflect higher visitor numbers that may occur on weekends or during the holiday season. These venues offered only drive-through and walk-through viewing options without direct contact. It is reasonable to assume that car traffic in venues P1 and P2 may be higher during peak periods.

##### Animal Management

Animal management scores were primarily within the low to intermediate range (Table 2 and Figure 3). A total of 38.2% of venues (*n* = 13) scored zero and 17.6% (*n* = 6) scored one, reflecting limited welfare understanding, premature cub removal, evidence of malnourishment or disease and extended use of lions in commercial activities. A further 38.2% of venues (*n* = 13) received a score of two, while only 5.9% (*n* = 2) scored three. No venues scored four, which would have indicated a strong understanding of animal welfare (Table 2).

Qualitative observations identified a range of management concerns, including frequent visitor tours, high tour volumes, provocative handling (e.g., V8 and V25), breeding activity and inadequate veterinary oversight (e.g., V5 and V30). Staff described large-scale breeding practices at some venues, including reports of up to 15 cubs born within two months, groupings of multiple breeding females (20 lionesses and 12 tigresses) and the breeding of hybrids, such as ligers (e.g., V5 and V8). Guides described trial-and-error approaches to safety and the use of food, sticks or verbal cues to move lions or position them for visitor photographs. Staff were observed encouraging lions to walk with visitors or to remain in specific positions during tours (e.g., V10 and V30).

Cubs were frequently handled for AVI activities over extended periods, including being carried or presented for petting (e.g., V5. V10. V26 and P9). Parasites, mange and poor body condition were observed in some cubs and adults, as well as signs of injury (e.g., fresh and old scars, and a subadult with a missing tail) (e.g., V5. V9. V20 and V27). Some facilities reported treating injuries or health concerns themselves without routine veterinary attendance (e.g., V5 and V18).

The two venues that scored three (P1 and V23) demonstrated comparatively stronger welfare management through the provision of veterinary care (explicitly addressed by V23), greater space and reduced commercial activities.

#### 3.2.3. Factor Analysis to Derive Single Measures of Welfare Across Venues

Two composite welfare variables were derived for subsequent analyses. For the analysis of welfare in relation to entry price, exploratory factor analysis was conducted across all ten welfare indices. A single underlying factor was identified, and internal consistency across the ten measures was high (Cronbach’s alpha = 0.84–0.87), indicating that the items reflected a common underlying construct of welfare.

For the analysis of welfare in relation to whether tourists were permitted to touch lions, a second factor analysis was conducted using nine welfare indices (excluding “entertainment”). This also yielded a single underlying factor, with similarly high internal consistency (Cronbach’s alpha = 0.80–0.85).

Welfare scores varied across venues in relation to the entry fee category (<ZAR100, ZAR100–250, ZAR250–500 and >ZAR500). Mean welfare scores (MWSs) were lowest for venues charging <ZAR100 (MWS = 9.35), followed by venues charging ZAR100–250 (MWS = 12.5), and highest for those charging ZAR250-500 (MWS = 15.6). No further increase in welfare scores was observed above ZAR500, and therefore the highest categories (ZAR250–500 and >ZAR500) were combined for analysis.

There was a positive association between entry fee category and welfare score (linear model: F = 4.616, d.f. = 1, *p* = 0.0416), indicating that higher entry fees were associated with improved welfare conditions. All welfare indices scores increased with entry fee.

Welfare scores also differed according to whether venues permitted AVI, with higher scores observed where AVI were not provided. The MWS was 13.01 at venues without AVI and 9.4 at venues offering AVI. This difference was statistically significant (linear model: F = 7.428, *p* = 0.0109), based on a model that excluded “entertainment intensity” from the welfare score derivation. Welfare scores were consistently higher across all indices at venues that prohibited direct contact between tourists and lions.

### 3.3. Other Felids

#### 3.3.1. Other Felids Species and Quantities

Nearly all venues (94.1%, *n* = 32) held other mammal species in addition to lions, while only 5.9% (*n* = 2) of venues held only lions. Across the 32 venues, a total of 607 individual captive animals from 21 mammal species were recorded. In addition, many bird and reptile species were observed but not systematically noted; hence, they, as well as any free roaming wild animals, were excluded from the total count. Venues held between 3 and 75 individual mammals over and above lions, with a mean of 18 individuals per venue, and between 1 and 9 mammal species per venue (median = 5 species per venue) (Table A2 and Table A3).

**Figure 4:**
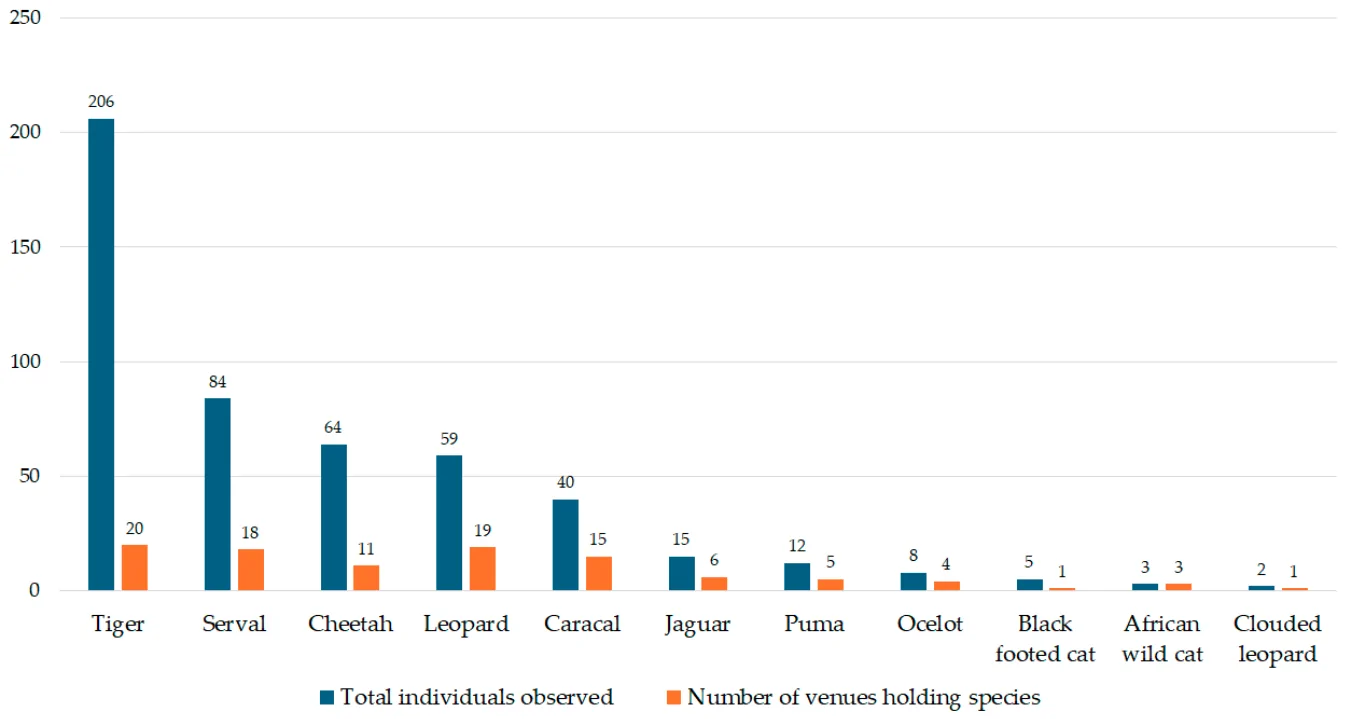
Total number of individual captive felids observed (blue) and the number of venues holding those individuals (orange) for each species across 30 venues that housed other felid species.

Of the 607 individual captive mammals recorded, 82.0% (*n* = 498) were other felids of 11 different species (Figure 4). As measured by the number of venues holding other felids, the most common species were tiger (*n* = 20 venues), leopard (*n* = 19 venues), serval (*n* = 18 venues) and caracal (*n* = 15 venues). The most frequently observed species by number of individuals were tiger (*n* = 206), serval (*n* = 84), cheetah (*n* = 64), leopard (*n* = 59) and caracal (*n* = 40). Black footed cat (*Felis nigripes*), African wild cat (*Felis lybica*) and Clouded leopard (*Neofelis nebulosa*) were the least frequently observed species (Figure 4 and Table A2).

Venues with higher welfare standards for lions were likely to hold fewer individuals of other felid species compared to those venues with lower welfare standards (ANOVA d.f. = 1, F = 12.197 *p* = 0.0014). The effect size was such that venues with welfare scores of 10.0 and under held a mean of 28.9 other felid individuals each, whereas those with welfare scores of 15.0 and greater held a mean of 8.2 individuals each. Similarly, there was evidence of an association between higher welfare standards and a lower diversity of other felid species held (ANOVA d.f. = 1, F = 4.1396, *p* = 0.050), such that venues with welfare scores of 10.0 and under held a mean of 4.6 other felid species, while those with scores of 15.0 or greater held a mean of 3.1 other felid species.

There was no evidence of an association between the entry price and the diversity of other felid species (ANOVA, d.f. = 1, F = 0.3970, *p* = 0.0574), or the total observed number of other felid individuals held (ANOVA d.f. = 1, F = 0.3326, *p* = 0.569). Additionally, there was no indication that venues allowing AVI were more likely to keep other captive wildlife species, host higher numbers of individuals, or exhibit a greater diversity of species.

#### 3.3.2. Welfare Conditions of Other Felids

Welfare conditions for other felids were broadly similar to those observed for lions. Practices involving AVI were common, with visitors entering enclosures or touching felids under staff direction. Adult AVI experiences involving serval were offered at 5.6% (*n* = 1) of the venues and cheetah at 27.3% (*n* = 3) of venues. Cub AVI experiences involving tiger were offered at 25.0% (*n* = 5) of venues, serval at 5.6% (*n* = 1) and caracal at 6.7% of venues (*n* = 1) (Table 4). A total of 69 tiger cubs and three serval kittens were observed during the assessment, indicating broader diversification and commercialisation of other felid species beyond lions.

Enclosure sizes for these felids were generally smaller than lion enclosures across the 30 venues that housed other felid species. However, some venues provided substantially smaller enclosures with, for example, three sub-adult leopards in 140 m^2^ (P5); one jaguar in 15 m^2^ (P8); and ocelot (*Leopardus pardalis*), leopard, jaguar, and puma in 50–60 m^2^ enclosures for two adults of each species (P9). Shade was provided, but in many cases not in adequate amounts. Poor hygiene and the absence of accessible drinking water were also occasionally observed, including the presence of old meat or bones in enclosures and dirty or green water sources (V22). While some venues offered more developed enrichment, such as tyres, pools or shaded corridors (V7), these were exceptions rather than the norm. Environmental complexity proved to be limited, with sparsely furnished enclosures containing only a few logs or a small shelter and lacking meaningful vegetation or cover.

Behavioural abnormalities, in particular pacing and fence-line walking (e.g., P8), were regularly observed across all five species, especially in enclosures with limited space or low structural complexity (Table 4).

Cub enclosures, particularly those housing tigers, contained virtually no natural features, consisting primarily of bare ground or minimal substrate variation (e.g., P3). Overall, many of these felids experienced restricted space, a lack of enrichment and handling regimes that limited behavioural expression and environmental comfort.

## 4. Discussion

### 4.1. Overview of Findings

A 2022 review of captive lion welfare highlighted the limited availability of empirical welfare data from commercial captive lion breeding facilities in South Africa, despite the scale of the industry [47]. Our study contributes to addressing this knowledge gap by providing systematic, facility-level welfare assessments across a large sample of tourism-orientated venues in South Africa holding lions and other felid species. The venues assessed catered to both local and international visitors, with common activities including predator viewing, cub petting and walking with lions. The majority of venues showed intermediate welfare conditions across all ten welfare indices, with hygiene and environmental noise quality receiving the highest scores overall; and animal management, enrichment, entertainment intensity and naturalness scoring the lowest overall. Importantly, none of the venues demonstrated consistently high scores across all ten welfare indices.

Most of South Africa’s provincial policies and augmenting legislation pertaining to captive lions primarily focus on the physical aspects of enclosure design, such as enclosure size and fencing standards, and to a much lesser degree on safety and welfare conditions [10]. Some provinces include generic requirements relating to basic welfare provisions, such as access to water supply and adequate shelter [10]. However, the qualitative analysis in the present study revealed systemic welfare concerns across all venues and multiple welfare indices. Common issues included restricted enclosure sizes, limited environmental complexity and enrichment, inadequate shelter and retreat opportunities, poor hygiene conditions, including dirty drinking water and accumulation of carcass remains, and inconsistent feeding practices. Husbandry conditions also varied across facilities and appeared to be influenced, in part, by differences in provincial legislation governing enclosure size and fencing requirements.

Overall, the welfare conditions of captive lions in tourist venues appeared to be constrained by the interaction between the physical environment and commercial husbandry practices. Structural, hygienic, and social stressors likely act together to restrict species-typical behaviour and may compromise long-term health and welfare outcomes.

### 4.2. Physical and Environmental Welfare Constraints

The physical environment is a key determinant of a captive animal’s daily experience. Across the 34 venues assessed, small enclosures and restricted mobility were common. While wild lions may occupy home ranges spanning hundreds of square kilometres, some captive adults were confined to areas as small as 250–300 m^2^. Such spatial restriction has been associated with musculoskeletal atrophy and stereotypic pacing [54,55], likely due to substantial reductions in opportunities for ranging or exploratory behaviours. Equally important is the qualitative design of enclosures. The absence of adequate shelter and retreat space limits behavioural choice and the sense of agency over the environment. A lack of controllability has long been recognised as an important contributor to chronic stress [56].

Poor hygiene conditions may further compound the welfare risks associated with spatial confinement and environmental restriction, while also increasing the risk of zoonotic disease transmission [32]. In wild populations, movement across large territories is likely to reduce the accumulation of parasites and pathogens within any single area [57]. In contrast, in confined environments, stagnant water sources and the retention of carcass material may increase opportunities for bacterial growth and parasite transmission. The observed cases of mange and poor body condition are therefore concerning, particularly in AVI venues where close proximity between tourists and lions is actively encouraged, raising additional concerns regarding zoonotic disease transmission [32].

### 4.3. Behavioural and Developmental Disruption

Behavioural and social disruptions were also observed, including early maternal separation being noted on account of the cubs’ young ages and the keeping apart from the mothers, artificial social groupings, signs of stress and aggression, and restricted opportunities for behavioural choice. Such patterns are consistent with other studies [22,23], which identified widespread concerns relating to behavioural abnormalities, nutritional deficiencies, and environmental stressors across captive settings. Disruptions to social and developmental processes to facilitate tourist activities were frequently reported; for example, cubs prematurely separated from their mothers within the first weeks of life. Such early separation is likely to disrupt important developmental periods, causing distress to both mothers and cubs, while also limiting opportunities for maternal care, antibody transfer, and early social learning [22,23]. Supporting this, a behavioural study across three tourism facilities reported that hand-reared cubs exhibited an abnormal activity budget, with cubs spending approximately 80% of their time inactive [58]. Unlike cubs in more natural environments, these individuals showed limited responsiveness to human stimuli, a pattern the authors associated with depressive-like states linked to maternal deprivation and compromised welfare in other mammals. Together, these findings suggest that early separation and intensive human handling may contribute to poor welfare outcomes.

Additional concerns relate to the physiological and behavioural consequences of hand-rearing itself. Cubs separated from their mothers shortly after birth may be deprived of colostrum, limiting early antibody transfer [22], while the absence of natural suckling opportunities may lead to the development of abnormal repetitive behaviours. In one facility (V5), hand-reared cubs were frequently observed engaging in non-nutritive sucking directed towards the ears and genitalia of conspecifics. This behaviour has been interpreted as frustration and stress associated with maternal deprivation and unmet suckling motivation [58].

Among the lions assessed in this study, most were housed in artificial social groupings, including with unrelated conspecifics or mixed species (e.g., lions housed with tigers), or at unusually high densities, which may contribute to elevated aggression or social instability.

### 4.4. Animal–Visitor Interactions and Welfare

Visitor experiences in the present study varied considerably across facilities, ranging from minimally supervised viewing to highly structured guided interactions involving continuous staff engagement and close animal contact. In venues where AVI was offered, overall welfare conditions were considerably lower. This finding is consistent with previous research demonstrating compromised welfare in lion cub AVI settings, including disrupted social development, stress-related behaviours and inappropriate husbandry practices [23,58]. Previous stakeholder-based research similarly identified the inability of cubs to retreat from interactions and the lack of control over their environment as among the most serious welfare concerns within lion interaction tourism settings [22].

The relationship identified between AVI tourism and reduced welfare is likely to reflect several interacting mechanisms. Tourism activities involving cub handling and close interactions typically require early cub separation, intensive habituation to humans, and management practices designed to maintain control and predictability during visitor encounters [22,58]. These conditions may restrict opportunities for behavioural choice and species-typical behaviour while increasing exposure to handling, noise and repeated disturbance. When animals are continuously exposed to visitors and mechanical noise without opportunities for withdrawal, as was observed in this study, the prolonged elevation of glucocorticoids can lead to systemic immune suppression and heightened vulnerability to disease [59,60,61,62]. Furthermore, observations made during the present study included repeated cub removals, staged interactions and instances of inappropriate handling, which may lead to chronic stress associated with persistent visitor exposure [63].

At a broader level, the positive relationship between entry price and animal welfare suggests that pricing structures may reflect underlying differences in business models, which is consistent with the relationship between production cost and the welfare of livestock [64]. Lower-cost venues may rely on higher visitor numbers and AVI experiences to generate revenue, potentially resulting in increased handling, noise, operational intensity and reduced opportunities for animals to withdraw from tourist exposure. In contrast, higher-cost venues may operate at lower visitor densities or rely more heavily on observational rather than AVI experiences, allowing for comparatively improved environmental conditions and husbandry practices. However, the relationship between price and welfare was not linear across all categories, and higher prices did not guarantee high welfare standards. No venue scored highly across all welfare indices, including the most expensive facilities. Entry price should therefore currently be interpreted as an imperfect proxy rather than a reliable indicator of acceptable welfare standards.

### 4.5. Commercial Drivers of Lion Welfare Outcomes

The patterns observed in this study should be considered within the broader context of the commercial captive predator industry in South Africa. Captive lion facilities often operate across multiple interconnected revenue streams, including tourism, captive hunting, breeding and the trade in live animals and derivatives [12,21]. Animal–visitor interactions, such as cub petting, bottle feeding, walking with lions and taking selfies, generate income while at the same time creating demand for a continued supply of young animals.

Within the broader commercial system, AVIs may also function as entry points into wider industry networks. Cubs used for AVI may later enter breeding programmes, captive hunting operations or other revenue streams once they are no longer suitable for public handling [10]. The welfare concerns identified in this study should therefore be understood not solely within the context of the tourism sector, but within a broader commercial framework, in which animals may move through multiple interconnected sectors over their lifespan.

### 4.6. Other Felids in Public-Facing Venues

Although this study focused primarily on lions, data recorded on other exotic and indigenous species revealed that 88.2% (*n* = 30) of the venues housed additional felids, totalling 498 individuals. This highlights the broader scope of commercial captive predator operations beyond lions alone and suggests that the welfare concerns identified in this study are not species-specific, but instead reflect systemic patterns across the wider captive predator industry.

South Africa’s provincial regulations for captive predators focus predominantly on lions, specifying minimum standards, such as minimum enclosure sizes that range from 120 m^2^ per lion in Gauteng to 10,000 m^2^ per lion in Mpumalanga, but there are generally no requirements for other felids [10]. As a result, we observed a range of very small enclosure sizes for a range of felids (Table 4). The prevalence of tigers is pertinent from a regulatory perspective. Tigers were present at more than half of the venues (58.8%, *n* = 20) and were the most numerous among the other felids (*n* = 206 individuals), indicating a substantial and potentially expanding market for exotic felids within this sector. As a non-indigenous species in the South African context, tigers occupy a comparatively ambiguous regulatory position and are subject to less sustained scrutiny than lions [30]. In the absence of integrated, multi-species regulation, there is a credible risk of regulatory displacement, whereby commercial breeding practices and associated welfare compromises are redirected from lions toward other, less-regulated felid taxa in response to target policy intervention. Any policy transition away from the commercial captive lion industry therefore needs to address this broader system directly, rather than risk the redistribution of welfare harms across related felid species.

### 4.7. Implications for South Africa’s Policy Direction

Following an extensive consultative process and majority recommendations of the High-Level Panel, the Minister of DFFE announced in May 2021 that South Africa intends to bring an end to the commercial captive lion industry. This commitment initiated the development of specific aspects of a policy framework, including the White Paper on the conservation and sustainable use of South Africa’s biodiversity (gazetted on 14 June 2023) and the Policy Position on the conservation and sustainable use of elephant, lion, leopard and rhinoceros (gazetted on 24 April 2024). Between May 2019 and November 2025, DFFE was led by ministers who engaged in formal policy processes to end the commercial captive lion industry. However, following a ministerial change in November 2025, no further policy developments have been instigated. For example, the Draft Notice Prohibiting Certain Activities Involving African Lions has progressed through all required processes, including approval by the National Council of Provinces, but has not been gazetted by the current minister at the time of writing.

In addition to policy development, an extended Ministerial Task Team (MTT) on the voluntary exit of the commercial captive lion industry developed training materials for Environmental Management Inspectorates (EMIs) to strengthen welfare-related enforcement capacity, although it has not yet been confirmed whether this training has commenced. This study reinforces existing evidence that animal welfare oversight remains constrained by limited institutional capacity, with both the national department and provincial authorities lacking sufficient capacity and expertise in animal welfare. These constraints are in part due to continued delays in implementing the MTT’s recommended welfare training [10,30]. This also includes insufficient resources and capacity to conduct regular monitoring inspections of captive lion facilities and delays in the renewal of expired permits [30]. Accordingly, a key recommendation from the MTT is to strengthen cooperation between the provincial nature conservation authorities and the NSPCA, including through regular joint EMI and NSPCA inspections of captive wildlife breeding and keeping facilities [10].

This study highlights systemic welfare deficits in South Africa’s public-facing captive lion venues; however, the majority of commercial captive predator facilities are fully off-grid farms without public access or facilities with an off-grid business component. Welfare conditions in these off-grid facilities are likely to be similar or potentially worse than those observed in public-facing venues [41]. Regulatory oversight across this sector remains fragmented and inconsistent across provinces, enforcement capacity is weak, and welfare standards are often governed through voluntary standards or weakly enforced mechanisms [10]. The widespread welfare concerns documented across 34 public-facing venues in this study suggest that existing regulatory frameworks may be insufficient to safeguard animal welfare within a commercially driven context. Similar concerns have been identified by AVI stakeholders, who reported the lack of governance and regulation as the single greatest welfare concern affecting lion AVI tourism in South Africa [22].

Taken together, our findings provide empirical support for the policy direction adopted by the South African government in 2021 to phase out its commercial captive lion industry. While improvements to regulatory oversight, enforcement capacity, and inter-agency collaboration remain important in the short term, the evidence presented in this study suggests that these measures alone are unlikely to address the systemic welfare challenges associated within the broader commercially driven captive lion industry. As such, the results are consistent with policy approaches prioritising a managed phase-out of this industry, alongside the development and promotion of wildlife-friendly tourism alternatives. Within this context, responsibly managed wildlife tourism models centred on the observation of lions and other felids within natural environments offer more ethically aligned wildlife experiences that avoid many of the welfare constraints inherent to close-contact captive tourism settings.

### 4.8. Limitations and Future Research

The welfare assessment framework applied in this study provided a structured and systematic approach for comparing welfare conditions across facilities by integrating multiple welfare domains into composite indices. The framework was intentionally designed around observable indicators and conditions accessible within tourism settings, enabling consistent assessments across a large sample of venues. However, as with most observational welfare frameworks, it cannot fully substitute for direct physiological measurements or longitudinal behavioural assessments, including chronic stress and long-term health outcomes. Future research incorporating longitudinal monitoring would provide additional insight into welfare conditions in response to seasonal, operational, or tourism-related changes.

Data collection was conducted through single visits to each venue and consequently reflects conditions at the time of assessment rather than longitudinal monitoring. Welfare conditions may vary over time, including during the peak tourism season. We acknowledge that single visits may not account for fluctuations in visitor numbers, feeding schedules, and associated varying levels of entertainment intensity, especially during peak holiday periods. Assessments were also limited to publicly accessible areas and have not captured conditions in restricted or off-display spaces, where management practices could differ. The use of covert observation further limited opportunities for detailed inquiry into husbandry protocols, veterinary care, and management records.

Further investigation of commercial off-grid facilities or facilities with an off-grid business component would provide additional understanding of welfare conditions across the wider captive predator industry. This is particularly important given that the majority of South Africa’s captive lion facilities are off-grid, without public access, where previous investigations have identified welfare concerns, including malnutrition, lack of clean water, inadequate shelter and enrichment, poor enclosure conditions, limited veterinary treatment and food restriction during periods of reduced hunting demand [24].

Nevertheless, structured observational approaches remain valuable tools for identifying welfare risks and broader comparative patterns within wildlife tourism settings, particularly where access to animals, veterinary records and management information is limited. The consistency of both the quantitative and qualitative findings across multiple independent welfare indicators strengthens confidence in the overall patterns identified in this study.

## 5. Conclusions

This study demonstrates that welfare conditions for captive lions in South African tourism venues are closely associated with commercial practices, particularly those involving AVI. The consistency of welfare deficits across multiple indices suggests that these concerns are systemic rather than isolated husbandry failures. Welfare conditions were also found to be compromised for other captive felid species that are currently not included within the policy decision-making processes associated with the potential phase-out of the commercial captive lion industry. This highlights the risk that policy change focused solely on captive lions may inadvertently shift commercial activities towards the other felids used in this industry. These findings therefore support the need for a managed phase-out of the commercial captive lion industry that also considers the potential displacement of commercial activities involving other captive felids and associated welfare harms.

## Figures and Tables

**Table 1:** Provincial distribution of total captive lion facilities (off-grid and public-facing) [7] and the proportion of venues assessed.

Province	Total Number of Facilities	Number of Venues Assessed	Venues Assessed as % of Total No. of Facilities
Eastern Cape	19	0	0%
Free State	131	9	6.9%
Gauteng	11	7	63.6%
KZN	2	0	0%
Limpopo	55	10	18.2%
Mpumalanga	6	0	0%
Northern Cape	0	0	0%
North West	93	8	8.6%
Western Cape	25	0	0%
**Total**	**342**	**34**	**9.9%**

**Table 2:** Description of the ten welfare indices and the five point score scale.

Category	Score 0	Score 1	Score 2	Score 3	Score 4
**Mobility**	Enclosures of <600 m^2^ with more than 5 lions, or <120 m^2^ per lion.	Intermediate of 0 and 2	Min. enclosure size of 2000 m^2^ up to 4 lions or plus 500 m^2^ per additional lion.	Intermediate of 2 and 4	Enclosures with min. 5000 m^2^ space per lion.
**Environmental Noise Quality**	Direct vicinity to main road or traffic, consistently large/noisy crowds, any noisy electronics (e.g., PA system, music).	Intermediate of 0 and 2	Occasional traffic or small visitor groups making noise, no large/noisy groups or electronic noise.	Intermediate of 2 and 4	No noise except natural sounds.
**Shelter**	Limited shelter external elements (sun, rain, etc.), barren/concrete ground.	Intermediate of 0 and 2	Some natural substrate areas, partial shelter options or adequate shelter but only enough for some lions.	Intermediate of 2 and 4	Natural ground substrate, sufficient and adequate shelter options for all lions.
**Hygiene**	Unhygienic enclosures (faeces, garbage, rotting food, dead animals).	Intermediate of 0 and 2	Relatively clean enclosures, possibly some remains of food and/or faeces from recent days.	Intermediate of 2 and 4	Frequent cleaning, no remains of food and/or faeces beyond most recent feedings.
**Naturalness**	Urban, barren, or fully artificial environment.	Intermediate of 0 and 2	Natural surroundings but predominantly artificial structures in the lion’s immediate vicinity.	Intermediate of 2 and 4	Fully functioning ecosystem.
**Social Interaction**	Overcrowding, long-term solitary housed lions, inappropriate sex ratio in enclosures or no visual barriers between conspecifics.	Intermediate of 0 and 2	Dense group housing with minimal opportunity for solitary retreat and enclosure features enabling visual barriers between conspecifics.	Intermediate of 2 and 4	Completely free choice of interaction or solitude with enclosure features to enable visual barriers between conspecifics.
**Enrichment**	No enrichment or structures in enclosures to provide vertical space.	Intermediate of 0 and 2	Some enrichment but limited in variety and not updated regularly. Minimal raised platforms.	Intermediate of 2 and 4	Abundance and variety of enrichment to encourage positive behaviours that aid physical and mental fitness with plenty of space and raised platforms.
**Diet Quality**	Inadequate amounts, limited variety, food depends entirely on entertainment activities dictated by tourists, inappropriate amounts of food supplemented by tourists, water bowls are dry or dirty.	Intermediate of 0 and 2	Water provided but not freely and is not frequently replenished/clean, food is given in adequate amounts but limited variety and inappropriate delivery.	Intermediate of 2 and 4	Sufficient food sources presented in enriching ways that allow free choice of consumption. Ad libitum water for all lions in enclosure.
**Entertainment Intensity**	Regular or intense commercial entertainment (selfies, feeding, walking with experiences).	Intermediate of 0 and 2	Limited commercial entertainment with ***no*** opportunity for lion’s voluntary withdrawal (selfies, feeding, walk with experiences).	Limited commercial entertainment with opportunity for lion’s voluntary withdrawal (selfies, feeding, walk with experiences).	No animal–visitor interactions or commercial entertainment experiences.
**Animal Management**	No welfare understanding, cubs separated from mothers prematurely, evidence of malnourishment or diseased, long working hours, primarily commercial interest.	Intermediate of 0 and 2	Moderate welfare understanding, some attempts to provide adequate captive conditions, limited working hours.	Intermediate of 2 and 4	Very strong welfare understanding and focus on best practise for lion welfare, resident vet or strong vet support, no exploitative commercial activities that impact the lions, sanctuary standard space and provisions.

**Table 3:** The offering of AVI (**a**), the provision of different types of information (**b**), and types of biosecurity measures (**c**) across the 34 assessed venues in South Africa.

(**a**)	**No AVI offered**	**No AVI policy**	**Petting**	**Feeding**	**Selfies**	**Walking**	**Provoking**
**Cub entertainment**	12	2	11	2	9	-	-
**Adult lion entertainment**			-	-	5	3	1
(**b**)	**Species**		**Conservation**		**Animal welfare**		
**Absent**	16		23		24		
**Basic**	11		5		7		
**Comprehensive**	7		6		3		
(**c**)	**Hand sanitizer**		**PPE**		**Disinfection**		
**Not provided**	24		21		17		
**Observed**	4		0		4		
**Unknown**	6		13		13		

**Table 4:** Summary of the welfare conditions observed for the five most common other felid species observed across 30 venues (*n* indicates number of venues).

	Tiger	Serval	Cheetah	Leopard	Caracal
**No of venues**	20	18	11	19	15
**Cubs available**	40.0%; *n* = 8 Total: 69 cubs	5.6%; *n* = 1 Total: 3 cubs	-	-	-
**Cub AVI offered**	25.0%; *n* = 5	5.6%; *n* = 1	-	-	6.7%; *n* = 1
**Adult AVI offered**	-	5.6%; *n* = 1	27.3%; *n* = 3	-	-
**Housed solitary**	45.0%; *n* = 9	22.2%; *n* = 4	18.2%; *n* = 2	36.8%; *n* = 7	40.0%; *n* = 6
**Mixed species**	15.0%; *n* = 3	-	-	-	-
**Enclosure not larger than 100 m^2^ for 4 adults**	-	5.6%; *n* = 1	-	-	6.7%; *n* = 1
**Poor hygiene**	20.0%; *n* = 4	11.1%; *n* = 2	-	5.3%; *n* = 1	6.7%; *n* = 1
**No shade**	-	-	-	-	-
**Limited shade**	35.0%; *n* = 7	33.3%; *n* = 6	54.5%; *n* = 6	21.1%; *n* = 4	26.7%; *n* = 4
**No accessible water**	5.0%; *n* = 1	11.1%; *n* = 2	9.1%; *n* = 1	5.3%; *n* = 1	13.3%; *n* = 2
**No submersible water**	25.0%; *n* = 5	N/A ^1^	N/A	N/A	N/A
**No enrichment**	20.0%; *n* = 4	-	18.2%; *n* = 2	10.5%; *n* = 2	6.7%; *n* = 1
**Underweight or overweight individuals**	4 overweight	-	1 overweight	1 underweight	3 overweight
**Injuries or deformities**	4 injuries	-	1 injury	-	2 injuries
**Behavioural abnormalities**	35.0%; *n* = 7	11.1%; *n* = 2	9.1%; *n* = 1	26.3%; *n* = 5	20.0%; *n* = 3

^1^ N/A as the absence or presence of submersible water for these species has no welfare implications since this is not part of their natural behaviour.

## Data Availability

The original contributions presented in this study are included in the article and Supplementary Material. Further inquiries can be directed to the corresponding author.

## Supplementary Materials

The following supporting information can be downloaded at: https://www.mdpi.com/article/10.3390/ani16172683/s1, File S1: Venue assessment form; File S2: Enclosures assessment form; File S3: Other felids assessment form.

## Appendix A

**Table A1:** The welfare assessment outcomes based on a five-point scoring system (0–4) for all ten welfare indices for the 34 tourism venues assessed in South Africa.

ID	Mobility	Noise	Shelter	Hygiene	Naturalness	Social	Enrichment	Diet	Entertainment	Management
V1	4	4	2	4	2	3	1	3	3	2
V2	4	4	4	4	3	3	3	2	3	2
V5	2	4	2	2	2	1	1	2	1	0
V6	2	2	3	3	2	3	2	2	1	0
V7	1	2	4	3	0	3	2	3	2	2
V8	2	2	4	4	2	2	2	3	2	1
V9	1	3	2	4	2	1	2	2	2	1
V10	1	2	2	3	2	2	2	2	0	0
V11	0	2	2	4	0	2	2	2	2	0
V12	3	2	3	4	3	3	1	2	3	2
V13	3	3	4	2	3	2	0	2	3	2
V17	2	3	2	3	2	2	1	1	0	0
V18	4	3	2	2	2	3	2	3	3	2
V19	3	2	1	2	1	1	0	2	2	0
V20	3	4	3	3	3	3	1	2	3	2
V21	3	3	2	4	2	2	2	3	3	2
V22	3	3	1	1	1	2	0	1	0	0
V23	4	2	4	4	3	4	3	3	3	3
V24	1	2	2	3	2	2	2	2	2	2
V25	1	3	2	3	2	2	2	2	2	1
V26	1	3	2	4	1	1	2	2	0	0
V27	4	3	3	3	3	4	2	2	3	2
V28	3	2	3	3	3	2	2	2	1	0
V29	4	3	4	1	3	4	4	3	3	2
V30	1	3	3	4	2	2	1	2	0	0
P1	4	3	4	4	3	4	2	3	3	3
P2	2	3	2	2	2	1	1	1	3	2
P3	2	2	3	3	2	3	2	2	2	1
P4	1	3	2	4	2	2	2	3	3	2
P5	1	2	2	2	2	2	1	1	2	0
P6	4	3	3	4	2	3	2	1	2	1
P7	4	2	2	4	2	3	2	2	1	1
P8	1	0	2	2	0	1	3	1	1	0
P9	0	2	0	1	0	0	0	1	0	0

**Table A2:** Other felid species and their numbers recorded at the 34 tourism venues assessed in South Africa.

ID	Tiger	Serval	Cheetah	Leopard	Caracal	Jaguar	Puma	Ocelot	Black Footed Cat	African Wild Cat	Clouded Leopard
V1		9			4						
V2		2									
V5	32			3		2					
V6					3			1		1	
V7		1	1				1				
V8	2	2		2	1			2		1	
V9	12	1		1	1		3			1	
V10	16	3		2	4						
V11	1			2	2						
V12	6	2		2	1						
V13	3	1	3	2							
V17		21									
V18				3							
V19		1			5						
V20											
V21	2	2		2							
V22											
V23	1			2			2				
V24	20				2						
V25	1					1					
V26	39	15	3	10	2	2	4				
V27											
V28	34	10		4	5						
V29				4							
V30	4	3	27	3	3						
P1			5								
P2	2	2	2	3		2		1			2
P3	3	2			2						
P4	4		2	2							
P5	2	6	1	6	4						
P6			5								
P7											
P8	1	1	2	2	1	2			5		
P9	21		13	4		6	2	4			
**Total number of felids**	**206**	**84**	**64**	**59**	**40**	**15**	**12**	**8**	**5**	**3**	**2**
**Total number of venues**	**20**	**18**	**11**	**19**	**15**	**6**	**5**	**4**	**1**	**3**	**1**

**Table A3:** Other mammal species and their numbers recorded at the 34 tourism venues assessed in South Africa.

	Hyena	Wolves	Wild Dog	Mongoose	Meerkat	Monkeys	Bat Eared Fox	Honey Badger	Jackal	Warthog
V1										
V2	2									3
V5					5	1				
V6	2	2				1	1	1		
V7		1								
V8								2		
V9	5			2	2					
V10										
V11	2									
V12	2		2				4			
V13	3		3			3			1	
V17										
V18										
V19			6							
V20										
V21										
V22										
V23	2	1					3			
V24		2								
V25		7							2	
V26										
V27	3									
V28							1	2		
V29										
V30	4									
P1										
P2										
P3	1		2							
P4										
P5		2								
P6	2									
P7	7		6							
P8										
P9	2		4							
**Total number of other** **mammals**	**37**	**15**	**23**	**2**	**7**	**5**	**9**	**5**	**3**	**3**
**Total number of venues**	**13**	**6**	**6**	**1**	**2**	**3**	**4**	**3**	**2**	**1**
