# Supplementary material for: Taking Stock: Assessing Animal Welfare in South African Captive Lion Tourism Venues
Source: Animals (Basel). 2026 Aug 27;16(17):2683. doi: 10.3390/ani16172683 (PMC13564327; doi:10.3390/ani16172683)
Supplement: Supplementary file 1 [file animals-16-02683-s001.zip › S1. Venue assessment form.pdf]

Venue Assessment form

Surveyor name: ____

Venue name: ____

Venue address: ____

Venue GPS: ____

Assessment date and time of visit: ____

Weather conditions during visit: ____

Venue

Opening days/hours: ____

Prices for (a) entry to facility (b) cub petting/selfies/feeding (c) walk with lions experience:

____

Number of daily visitors & source of this information: ____________________________

○ Set number of tours per day ○ Tours on demand (Average # tours per day): ___________

Education resources (relating to lions, specifically):

[Basic info = e.g., posters, signage / Comprehensive info = e.g., obligatory videos, dedicated staff, lectures]

Species information: ○ No info ○ Basic info ○ Comprehensive info
Conservation: ○ No info ○ Basic info ○ Comprehensive info
Animal welfare: ○ No info ○ Basic info ○ Comprehensive info
Other: ○ No info ○ Basic info ○ Comprehensive info

Basic biosecurity & safety resources:

Physical interaction between animals and people allowed (including walking with): ○ Yes ○ No

Hand sanitizer (staff/visitors*): ○ Observed ○ Optional ○ Not provided ○ Unknown

PPE & footwear for enclosure (staff/visitors*): ○ Observed ○ Optional ○ Not provided ○ Unknown

Disinfection on arrival/exit of enclosure (staff/visitors*): ○ Observed ○ Optional ○ Not provided ○ Unknown
* *Delete as appropriate*

Unprotected (without barrier): ○ Staff ○ Visitors ○ Both ○ Nobody
Any safety measures in place: ○ Staff ○ Visitors ○ Both ○ Nobody
If so, describe safety measures provided for direct contact with lions:

Adults:____

Cubs:____

Lions:
Total lions at facility: ____
Reported by venue: Male: __________Female: ____________Cubs: _____________
Observed by surveyors: Male: ____________ Female: ______________ Cubs: _____________
No. of white lions observed: ____
No. of cross-bred lion species (e.g. ligers) observed: ______________________________

Health and behaviour

Veterinary care: ○ Vet on site ○ External vet on demand ○ Keeper-lead treatment ○ Unknown

Frequency of health checks by trained vet: ○ Daily ○ Weekly ○ Monthly ○ Yearly ○ Unknown

Noise levels surrounding enclosures (dB): _______________ ☐ Noisy ☐ Moderate ☐ Quiet

No. lions visibly (A) underweight (B) overweight: A:________________B:_________________________

No. lions with signs of (A) disease (B) injuries: A:__________________B:_________________________

No. lions displaying stereotypical or behavioural abnormalities: ________________________________

No. lions with physical deformities that could indicate inbreeding: ______________________________

Add details in the notes on any of the above.

Enclosures: *(Please also fill out "Enclosure assessment form")*

Total number of lion enclosures: ____

Number of lions kept on chains or leashes:____

Number of lions housed solitary: ____

Number of enclosures with > 4 adult lions: ____

Number of cubs < 6 months old, housed with all cubs (no adults): _____________________________

Number of enclosures housing lions together with other cat species: ___________________________

Number of adult lions in enclosures < 50 m^2: ____

Number of adult lions in enclosures 50 m^2 – 120 m^2: ____

Number of adult lions in enclosures 120 m^2 – 400 m^2: ____

Number of adult lions in enclosures 400 m^2 – 2,500 m^2: _______________________________________

Diet:

Feeding frequency per week: ○ 1 ○ 2 ○ 3 ○ 4 ○ 5 ○ 6 ○ daily ○ Unknown

Delivery: ○ *All* feeding coincides with interaction experiences / guided tour*

○ *Some* feeding coincides with interaction experiences / guided tour*

○ Feeding is not dependent on interactive experience / guided tour*

○ Unknown

* *Delete as appropriate.*

Are snacks available for visitors to purchase and feed lions? ○ Yes ○ No

Details on type of meat and delivery:

(include whether prey is provided dead or alive, as morsels or as whole carcass, and if any additional supplemental vitamins or minerals are provided):

____

____

Breeding

Does facility breed lions? ○ Yes ○ No – never have ○ No – have stopped in recent years

Is breeding controlled? If so, how? (e.g., single sex housing, contraception, sterilisation)

____

Number of cubs born at facility per year (average): _______________________________________

At what age are cubs separated from their mother?____

Entertainment:

Facility offers (tick all that apply):

☐ Self-guided tours ☐ Guided tours ☐ Walk through ☐ Game drive

☐ Viewing lions on foot behind a fence ☐ Viewing lions from vehicle inside enclosure

☐ Cub petting ☐ Cub feeding by visitors ☐ Cub selfies (inside enclosure)

☐ Entertainment feeding (staff feed lions on demand for entertainment, more than husbandry feeding)

☐ Young/adult lion 'walk with' experiences ☐ Young/adult lion selfies

☐ Lion performances or shows

☐ Staff provoke lions for tourist entertainment

☐ Facility has clear policy against lions for entertainment

Number of interactive experiences offered per day:

○ < Once daily ○ Once daily ○ Twice daily ○ 3+ ○ 6+ ○ On demand no limit ○ Other

Average length of interaction with cubs:

○ < 15 minutes ○ 15 – 30 minutes ○ > 30 minutes

Average length of interaction with young/adult lions:

○ < 15 minutes ○ 15 – 30 minutes ○ > 30 minutes

Number of lions available for interactive experiences:

Adults: ____________________________ Cubs: _____________________________

General description of venue after assessment and other notes:

____
____
____
____
____
____
____
____
____
____
____
____
____
____
____
____
____
____
____
____
____
____

Based on the overall conditions at the venue (that apply to the majority of lions/enclosures), please provide a score for the following categories, based on the information table below:

**Mobility**	○ 0	○ 1	○ 2	○ 3	○ 4
**Environmental noise quality**	○ 0	○ 1	○ 2	○ 3	○ 4
**Shelter**	○ 0	○ 1	○ 2	○ 3	○ 4
**Hygiene**	○ 0	○ 1	○ 2	○ 3	○ 4
**Naturalness**	○ 0	○ 1	○ 2	○ 3	○ 4
**Social interaction**	○ 0	○ 1	○ 2	○ 3	○ 4
**Enrichment**	○ 0	○ 1	○ 2	○ 3	○ 4
**Diet quality**	○ 0	○ 1	○ 2	○ 3	○ 4
**Entertainment intensity**	○ 0	○ 1	○ 2	○ 3	○ 4
**Animal management**	○ 0	○ 1	○ 2	○ 3	○ 4

**Category**	**0**	**1**	**2**	**3**	**4**
Mobility	< 600 m^2 with more than 5 lions, or < 120 m^2 per lion.	Intermediate of 0 and 2	Min. 2,000 m^2 up to 4 lions or plus 500 m^2 per additional lion	Intermediate of 2 and 4	Enclosures with min. 5,000 m^2 space per lion
Environmental noise quality	Direct vicinity to main road or traffic, consistently large/noisy crowds, any noisy electronics (e.g., PA system, music)	Intermediate of 0 and 2	Occasional traffic or small visitor groups making noise, no large/noisy groups or electronic noise	Intermediate of 2 and 4	No noise except natural sounds
Shelter	limited shelter external elements (sun, rain, etc…), barren/concrete ground	Intermediate of 0 and 2	Some natural substrate areas, partial shelter options or adequate shelter but only enough for some lions	Intermediate of 2 and 4	Natural ground substrate, sufficient and adequate shelter options for all lions
Hygiene	Unhygienic enclosures (faeces, garbage, rotting food, dead animals),	Intermediate of 0 and 2	Relatively clean enclosures, possibly some remains of food and/or faeces from recent days	Intermediate of 2 and 4	Frequent cleaning, no remains of food and/or faeces beyond most recent feedings
Naturalness	Urban, barren, or fully artificial environment	Intermediate of 0 and 2	Natural surroundings but predominantly artificial structures in lion's immediate vicinity	Intermediate of 2 and 4	Fully functioning ecosystem
Social interaction	Overcrowding, long-term solitary housed lions, inappropriate sex ratio in enclosures,	Intermediate of 0 and 2	Dense group housing with minimal opportunity for solitary retreat,	Intermediate of 2 and 4	Completely free choice of interaction or solitude.
Enrichment	No structures to provide vertical space or visual barriers between conspecifics	Intermediate of 0 and 2	Minimal raised platforms or enclosure features enabling visual barriers between conspecifics	Intermediate of 2 and 4	Plenty of space, raised platforms and enclosure features to enable visual barriers between conspecifics
Diet quality	Inadequate amounts, limited variety, food depends entirely on entertainment activities dictated by tourists, inappropriate amount of food supplemented by tourists, water bowl is dry or dirty	Intermediate of 0 and 2	Water provided but not freely and is not frequently replenished/clean, food is given in adequate amounts but limited variety and inappropriate delivery	Intermediate of 2 and 4	Sufficient food sources presented in enriching ways that allows free choice of consumption. Ad-libitum water for all lions in enclosure

Entertainment intensity	Regular or intense commercial entertainment (selfies, feeding, walk with experiences)	Intermediate of 0 and 2	Limited commercial entertainment with ***no*** opportunity for lion's voluntary withdrawal (selfies, feeding, walk with experiences)	Limited commercial entertainment with opportunity for lion's voluntary withdrawal (selfies, feeding, walk with experiences)	No animal-visitor interactions or commercial entertainment experiences.
Animal Management	No welfare understanding, cubs separated from mothers prematurely, evidence of malnourishment or diseased, long working hours, primarily commercial interest	Intermediate of 0 and 2	Moderate welfare understanding, some attempts to provide adequate captive conditions, limited working hours	Intermediate of 2 and 4	Very strong welfare understanding and focus on best practise for lion welfare, resident vet or strong vet support, no exploitative commercial activities that impact the lions, sanctuary standard space and provisions

Additional species

Please detail all other species that are observed and/or reported at the venue. Please capture images of each species as evidence.

**Species**	**Number reported**	**Number observed**	**Corresponding image ID**
