# Supplementary material for: Taking Stock: Assessing Animal Welfare in South African Captive Lion Tourism Venues
Source: Animals (Basel). 2026 Aug 27;16(17):2683. doi: 10.3390/ani16172683 (PMC13564327; doi:10.3390/ani16172683)
Supplement: Supplementary file 1 [file animals-16-02683-s001.zip › S2. Enclosures assessment form.pdf]

Enclosure assessment form (complete one per different style enclosure)

Surveyor name: ____

Venue name: ____

Enclosure number: ____

Enclosure design

Barrier (tick all that apply):

☐ Metal/wire mesh fence ☐ Gaps in mesh < 10 x 10 cm ☐ Partial electric fence / electrified wires
☐ Enclosure fence < 2.4m high ☐ Enclosure fence 2.4 - 4.5m high ☐ Enclosure fence > 4.5m high
☐ Inside overhang on fence
☐ Double barrier ☐ Padlock gates ☐ Airlocks ☐ Roof structure ☐ Alarm system
☐ Hazardous animal signage ☐ Moat (water or dry substrate) ☐ Fire hose
☐ Secure space for management access to enclosure
☐ If enclosure contains > 4 lions, is the area > 2500 m² & the fence > 5m high

Stand-off space between external enclosure barrier and public: ○ Yes ○ No

Ground substrate (tick all that apply):

☐ Concrete/hard surface ☐ Dirt ☐ Grass ☐ Wood ☐ Sand ☐ Straw ☐ Other

Hygiene (tick all that apply during visit):

☐ Clean area ☐ Moist/wet ground ☐ Dry ground ☐ More than day old faeces ☐ Urine stench
☐ Garbage ☐ Rotten food ☐ Old bones ☐ Deceased animals

Enrichment (tick all that apply):

☐ Multi-level climbing structures/elevated platforms ☐ Scratching posts / trees ☐ Logs
☐ Termite mounds ☐ Manipulable objects (boxes, bark, hay grasses, skin from other animals, balls)
☐ Chewing items ☐ Hanging items (ropes, branches)
☐ Bathing area ☐ Scent enrichment
☐ Hidden or suspended food
☐ Other (describe in notes)
☐ No evidence of enrichment

How often is enrichment provided/renewed (according to venue staff):
○ Daily ○ Weekly ○ Monthly ○ Other ○ Unknown

Enclosures (social interaction)

Size of enclosure: ____
Number of females: ____
Number of males:____
Number of cubs < 6 months old: ____

Provisions (tick all that apply):

☐ Partial shaded area (single tree, mesh net) with available space for *some* lions in enclosure
☐ Partial shaded area (single tree, mesh net) with available space for *all* lions in enclosure
☐ Full shaded area (roof, dense canopy) with available space for s*ome* lions in enclosure
☐ Full shaded area (roof, dense canopy) with available space for *all* lions in enclosure
☐ No access to direct sunlight ☐ Indoor area available ☐ Den area available
☐ Visual barriers available to hide from tourist view
☐ Hiding areas available for escaping conspecifics
☐ Continuous/unlimited access to water for drinking (e.g. hose, feeder)
☐ Water bowl (fresh water) ☐ Water bowl (dirty water only) ☐ Water bowl (dry, no water)
☐ Access to water for submersion (river/pond)

Any other points to note (about enclosures):

____
____
____
____
____
____
____
____
____
____
____
____
____
____
____
____
____
____
____
____
____
____
____
