# Supplementary material for: Taking Stock: Assessing Animal Welfare in South African Captive Lion Tourism Venues
Source: Animals (Basel). 2026 Aug 27;16(17):2683. doi: 10.3390/ani16172683 (PMC13564327; doi:10.3390/ani16172683)
Supplement: Supplementary file 1 [file animals-16-02683-s001.zip › S3. Other felids assessment form.pdf]

Felid focussed assessment form

Surveyor name: ____

Venue name/date of visit: ____

	Felids									
	Leopard	Cheetah	Caracal	Serval	Black footed cat	Tiger	Snow leopard	Jaguar	Cougar	Puma
No. adults observed at venue										
No. cubs observed at venue										
(Y/N) Cubs offered in direct interactions with visitors or as part of performance?										
(Y/N) Adults offered in direct interactions with visitors or as part of performance?										
(Y/N) Housed in enclosures with other species?										
(Y/N) Housed in enclosures < 100 m^2 for 4 adults										
(Y/N) Housed solitary?										
(Y/N) Housed in enclosures with poor hygiene? (use hygiene indicators from lion form)										
(Y/N) Housed in enclosures without shade?										

(Y/N) Housed in enclosures with limited shade?										
(Y/N) Housed in enclosures without freely accessible water?										
(Y/N) Housed in enclosures without enrichment? (use enrichment indicators from lion form)										
(Y/N) Housed in enclosures without access to water for submersion?										
(Y/N) Housed in enclosures without visual barriers to retreat from public view?										
(Y/N) Visibly underweight?										
(Y/N) Visibly overweight?										
(Y/N) Evidence of injuries or physical deformities?										
(Y/N) Evidence of behavioural abnormalities ?										

Notes:

____
____
____
____
____
____

____
____
____
____
____
____
____
____
____
____
____
____
____
____
____
____
____
____
____
